# In-patient outcomes of Hematopoietic Stem Cell Transplantation in Patients with Immune Mediated Inflammatory Diseases: A Nationwide Study

**DOI:** 10.1038/s41598-018-24060-4

**Published:** 2018-05-01

**Authors:** Kathan Mehta, Palashkumar Jaiswal, Farren Briggs, William A. Faubion, James H. Tabibian, Fabio Cominelli, Maneesh Dave

**Affiliations:** 1 Division of Hematology and Oncology, University of Pittsburgh Medical Center, Pittsburgh, Pennsylvania USA; 2 Department of Internal Medicine, John H Stroger, Jr. Hospital of Cook County, Chicago, Illinois USA; 3 Department of Epidemiology and Biostatistics, Case Western Reserve University, Cleveland, Ohio USA; 4 Division of Gastroenterology and Hepatology, Mayo Clinic, Rochester, Minnesota USA; 5 Division of Gastroenterology, Department of Medicine Olive View-UCLA Medical Center, Sylmar, CA United States; 6 Division of Gastroenterology and Liver Disease, University Hospitals, Case Western Reserve University, Cleveland, Ohio USA

## Abstract

The impact of underlying immune-mediated inflammatory diseases (IMID) in patients undergoing hematopoietic stem cell transplant (HSCT) is unclear. Hematopoietic cell transplantation co-morbidity index (HCT-CI) is gaining acceptance as a reliable clinical method to score pre-transplant co-morbidities. Higher HCT-CI from a co-morbid IMID implies higher NRM. However, HCT-CI integrates many IMIDs with different pathogenesis and treatment together which may lead to spurious results. We performed a cross-sectional study using Nationwide Inpatient Sample dataset from 1998 to 2011 to compare the outcomes of HSCT in patients with different co-morbid IMIDs with patients without any co-morbid IMIDs. In both our multivariate and stringent matched-pair analysis, ulcerative colitis (UC) was associated with increased mortality while rheumatoid arthritis and psoriasis were associated with lower mortality as compared to no IMID group. Furthermore, in allogeneic HSCT subgroup, UC was associated with higher mortality and psoriasis was associated with lower mortality. In conclusion, we found that depending on the type of HSCT, each IMID has a different impact on outcomes of HSCT. Furthermore, UC patients had increased mortality if they had primary sclerosing cholangitis and had a higher risk of opportunistic infections like tuberculosis and cytomegalovirus suggesting the need for increased vigilance in this cohort.

## Introduction

Hematopoietic stem cell transplantation (HSCT) involves infusion of hematopoietic stem cells to treat malignant (e.g. leukemia) as well as non-malignant (e.g. aplastic anemia) hematologic and lymphoid diseases^1^. HSCT involves conditioning regimens, i.e. radiation and chemotherapy, which places patients at increased risk of complications (e.g. opportunistic infections [OI’s]) and mortality^2–4^. Recent cumulative evidence suggests that transplant related mortality (TRM) varies between 8–36% depending on the indication for HSCT, age group and type of HSCT^5–7^. Certain underlying co-morbid conditions can further add to the morbidity and mortality of patients undergoing HSCT^8^.

Immune-mediated inflammatory diseases (IMIDs) constitute the third most common chronic medical condition and have an estimated prevalence between 5% to 8% in the United States (US) population alone^9^. Moreover, IMIDs are one of the top 10 causes of death in women in the US^10^. Inflammatory bowel diseases (IBD), rheumatoid arthritis (RA), psoriasis, systemic lupus erythematosus (SLE), multiple sclerosis (MS), systemic sclerosis (SS) and diabetes mellitus type I (DM I) are among the most commonly observed IMIDs in the US with increasing incidence and prevalence^11–14^. With the increase in prevalence of IMIDs and the rise in the number of HSCT’s performed in US^15^, many more patients undergoing HSCT are expected to have a co-morbid IMID. The pathogenesis of IMIDs involves aberrations in interaction of adaptive and innate immune system with environmental and microbiological factors^16–19^. Thus a co-morbid IMID may alter the risk of graft-versus-host-disease (GVHD) and infection in patients undergoing HSCT^20^. In a retrospective study Sorror *et al*., in 2005, demonstrated that co-morbid IMIDs like IBD, rheumatological disease (defined as RA, SLE, mixed connective tissue disorder, polymyositis and polymyalgia rheumatica) and diabetes were independently associated with significantly increased non relapse mortality (NRM) in patients who underwent allogeneic HSCT for treatment of malignant or non-malignant hematological conditions^8^. The authors developed a Hematopoietic Cell Transplantation Comorbidity Index (HCT-CI) that included IMIDs like IBD and rheumatological diseases and which predicted NRM in HSCT patients. NRM was higher in patients with higher HCT-CI. In a follow up multi-institutional retrospective study in 2014, Sorror and colleagues demonstrated that higher HCT-CI (included patients with IMIDs) is associated with higher risk of developing acute GVHD as well^20^. Furthermore, comorbidities such as IBD and rheumatological diseases were found to independently increase the risk of death^8^ and acute GVHD^20^. However, Rabian *et al*., in a recent retrospective case-control analysis that included 18 IBD patients and 50 controls who underwent allogeneic HSCT for hematologic conditions, did not find any significant difference in cumulative incidence of acute GVHD or NRM between IBD and control group^21^. The first two studies show that mortality is increased with IMID like IBD and rheumatological diseases and the third did not find increased mortality with IBD. These three studies integrated many IMIDs together including IBD that comprises of ulcerative colitis (UC) and Crohn’s disease (CD) when they reported outcomes. IMIDs albeit immune mediated, have different pathogenesis, natural history and management and integrating them together can lead to spurious results^22–27^. Hence, the impact of individual IMIDs in patients undergoing HSCT requires further investigation. The aim of our study was to determine in-hospital mortality, complications, length of stay (LOS), and costs associated with HSCT in patients with co-morbid IMIDs compared to patients without IMIDs in US using a nationwide database and a stringent matching methodology.

## Results

From 1998 to 2011, the NIS contained data on more than 8 million discharges each year. Among this group there were 36,166 patients who underwent HSCT; this is representative of 182,166 similar patients across US after weighting. Out of 36,166 patients who underwent HSCT, UC, CD, RA, SLE, MS, psoriasis, DM I, and SS were the concurrent IMID subgroups with sample size of at least 50 patients per group. Since the number of patients for other IMIDs was less than 50, they were not included in the analysis. The patients who had multiple IMIDs from the above list (n = 12) were also excluded from the analysis. The final dataset contained 36,154 (weighted 182,107) patients. In matched pair analysis, only 9 patients with SS matched with controls, therefore SS was excluded from detailed analysis. The cohort of IMID patients included a total of 694 patients, with 78 patients in the UC group, 72 patients in the CD group, 137 patients in the RA group, 99 patients in the psoriasis group, 74 patients in the SLE group, 143 patients in the DM I group and 91 patients in the MS group as compared to 35,460 patients in the no IMID group. 46.7% of patients in the IMID group were male as compared to 56.7% in the no IMID group. The most common source of the stem cells in this study was peripheral blood which was similar to reported in other studies^28,29^ (Table 1). Additional baseline characteristics of the patients are shown in Table 1 and Supplementary Table S1. We also did subgroup analyses in patients undergoing allogeneic HSCT with and without co-morbid IMID for the same outcomes (Table 2). We have presented the data on outcomes of HSCT in patients with co-morbid IMIDs from multivariable analyses in Tables 1 and 2, from matched pair analyses in Table 3, and from multivariate logistic regression analysis to identify predictors of mortality in patients undergoing HSCT in Table 4.

**Table 1:** Baseline Characteristics and Outcomes in Patients Undergoing HSCT from 1998 to 2011.

Baseline characteristics	UC	CD	RA	Psoriasis	SLE	DM I	MS	No IMID
Actual (projected) number of HSCT in the US	78 (391)	72 (367)	137 (684)	99 (497)	74 (373)	143 (720)	91 (452)	35,460 (178,624)
**Patient characteristics**								
Age	46.05 ± 2.07	49.33 ± 1.76	56.42 ± 0.97	50.66 ± 1.43	44.14 ± 1.78	46.80 ± 1.24	42.88 ± 1.30	42.95 ± 0.10
Male (%)	60.4	57.0	40.8	68.6	9.8	54.6	31.5	56.7
Race (%)								
White	68.3	66.1	66.7	73.4	49.3	53.5	62.9	59.1
Non-white	18.7	9.1	14.6	9.3	19.5	26.5	9.7	20.5
Missing	13.0	24.8	18.7	17.3	31.2	20.0	27.5	20.4
CCI^a^	2.34 ± 0.17	1.71 ± 0.15	3.05 ± 0.11	2.25 ± 0.08	2.72 ± 0.17	3.05 ± 0.08	1.10 ± 0.15	2.24 ± 0.01
Type of HSCT								
Allogeneic (%)	46.8	51.0	33.7	37.5	35.2	44.7	21.4	41.5
Autologous (%)	53.2	47.8	66.3	62.5	63.6	54.6	78.6	58.2
Stem cell source								
Peripheral Blood	82.9	83.8	90.6	92.3	85.8	75.1	89.1	80.4
Bone Marrow	13.3	13.5	7.9	7.7	11.4	22.8	8.7	16.6
Cord blood	3.7	2.7	1.5	0.0.	2.7	2.1	2.2	3.0
Conditioning								
TBI^a^	14.1	14.1	7.3	7.2	8.4	13.1	18.6	13.2
No TBI^a^	85.9	85.9	92.7	92.8	91.6	86.9	81.4	86.8
**Outcomes:**								
**Length of Stay (Days), Total Charges (in Thousand $), Complications (%) and in-Hospital Mortality (%)**								
LOS^a^ (Mean ± SE)	34.3 ± 2.6**	25.8 ± 1.4	21.4 ± 0.9***	22.6 ± 1.0***	25.4 ± 1.5	27.7 ± 1.6	21.2 ± 0.9***	26.8 ± 0.1
Total charges (Mean ± SE)	344.6 ± 38.1**	234.5 ± 16.9	184.3 ± 14.3 ***	197.3 ± 12.9 *	272.4 ± 27.3	228.3 ± 14.1	173.8 ± 7.9***	231.8 ± 1.1
FN^a^	36.7	33.3	35.2	41.8*	38.7	25.1*	44.2	32.6
Bacteremia	32.0**	21.6	11.6*	10.2**	15.4	19.0	8.4*	19.2
ODI^a^	46.9**	34.4	22.3*	31.2	30.6	37.2	29.6	32.9
OI’s^a^	30.8***	13.0	6.5*	12.1	11.7	13.8	13.3	13.4
GVHD^a^	17.0***	0.0***	4.1	8.2	3.0*	2.5*	2.2*	7.7
Stomatitis	42.0	43.0	41.7	47.3	23.4**	39.2	27.2	46.0
TPN^a^	18.8	16.0	11.7	9.2*	9.7	19.4	11.2	17.5
Intubation	12.4**	4.5	1.4***	0.0**	6.7	6.4	3.1	4.2
Death (%)	16.5***	3.3	1.3*	0.0***	6.8	9.3	3.3	5.8

^a^Abbreviations: IMID, Immune mediated Inflammatory disease; CCI, Charlson Co-morbidity Index; TBI, Total Body Irradiation; ODI, Other Documented Infections; OI’s, Opportunistic infections; GVHD, Graft-vs.-Host Disease, available after 2007; TPN, Total Parenteral Nutrition; LOS, Length of Stay; FN, Febrile Neutropenia; HSCT, Hematopoietic Stem Cell Transplantation; UC, Ulcerative Colitis; CD, Crohn’s Disease; RA, Rheumatoid Arthritis; DM, Diabetes Mellitus; SLE, Systemic Lupus Erythematosus; MS, Multiple Sclerosis.

For outcomes: *P ≤ 0.05 as compared to no IMIDs group; **P ≤ 0.01 as compared to no IMIDs group; ***P ≤ 0.001 as compared to no IMIDs group.

**Table 2:** Baseline characteristics and outcomes in patients undergoing Allogeneic HSCT from 1998 to 2011.

Baseline characteristics	UC	CD	RA	Psoriasis	SLE	DM-I	MS	No IMIDs
Actual (projected) number of HSCT in the US	37 (183)	37 (187)	46 (230)	37 (186)	26 (131)	64 (322)	19 (97)	14,701 (74,189)
**Patient Characteristics**								
Age	40.15 ± 3.00	46.05 ± 2.61	54.62 ± 1.64	46.54 ± 2.39	42.45 ± 2.41	42.75 ± 1.95	48.19 ± 2.88	37.80 ± 0.16
Male (%)	58.06	65.11	39.28	76.06	3.98	61.19	35.28	58.13
Race (%)								
White	67.56	75.67	71.73	72.97	61.53	62.50	78.94	57.28
Non-white	26.47	8.10	13.04	8.10	11.50	20.31	0.00	21.37
Missing	5.96	16.22	15.22	18.92	26.92	17.18	21.05	21.34
CCI	2.03±0.25	1.69±0.24	2.56±0.16	2.10±0.15	2.65±0.38	2.81±0.13	1.75±0.22	1.94±0.01
Source of stem cell								
Peripheral blood	71.61	79.11	76.05	84.57	77.93	64.12	64.50	64.15
Bone marrow	20.42	15.56	19.44	15.42	14.34	31.07	25.27	28.62
Cord blood	7.95	5.31	4.49	0	7.71	4.79	10.22	7.22
Conditioning								
TBI	21.77	25.04	15.36	10.90	16.03	20.29	23.46	24.55
No TBI	78.28	74.94	84.63	89.09	83.96	79.70	76.53	75.44
**Outcomes:**								
**Length of Stay (Days), Total Charges (in Thousand $), Complications (%) and in-Hospital Mortality (%)**								
LOS^a^ (Mean ± SE)	45.1 ± 4.3*	27.1 ± 2.1***	25.0 ± 2.5***	25.5 ± 2.3***	33.6 ± 3.2	35.4 ± 2.9	26.8 ± 2.5 **	35.5 ± 0.2
Total charges (Mean ± SE)	513.7 ± 67.4***	281.8 ± 24.2*	262.4 ± 38.7*	267.2 ± 27.3*	428 ± 62.8	309 ± 24	221.3 ± 19.3***	335.1 ± 2.1
FN^a^	30.0	24.1	33.6	41.0	18.6	24.0	14.6	29.4
Bacteremia	40.3*	16.7	10.9	5.1**	24.9	22.7	15.1	23.6
ODI^a^	55.9	31.0	25.7*	40.5	53.8	43.8	52.0	41.0
OI’s^a^	43.6***	8.7	10.9	21.1	21.9	20.2	31.5	20.2
GVHD^a^	29.6**	0.0***	10.7*	20.3	10.2	5.6**	11.5	17.2
Stomatitis	30.4	51.7	27.5*	42.8	27.4	45.6	32.3	45.4
TPN^a^	27.2	28.6	15.4	16.4	15.9	27.6	12.6	23.4
Intubation	19.0*	8.9	2.0	0.0	19.1*	6.5	0.0***	6.9
Death (%)	24.1*	6.5	4.0	0.0***	19.2	9.9	10.7	10.7

^a^Abbreviations: IMID, Immune mediated Inflammatory disease; CCI, Charlson Co-morbidity Index; TBI, Total Body Irradiation; ODI, Other Documented Infections; OI’s, Opportunistic infections; GVHD, Graft-vs.-Host Disease, available after 2007; TPN, Total Parenteral Nutrition; LOS, Length of Stay; FN, Febrile Neutropenia; HSCT, Hematopoietic Stem Cell Transplantation; UC, Ulcerative Colitis; CD, Crohn’s Disease; RA, Rheumatoid Arthritis; DM, Diabetes Mellitus; SLE, Systemic Lupus Erythematosus; MS, Multiple Sclerosis

For outcomes: *P ≤ 0.05 as compared to no IMIDs group; **P ≤ 0.01 as compared to no IMIDs group; ***P ≤ 0.001 as compared to no IMIDs group.

**Table 3:** Outcomes of HSCT in Matched Sample of Patients with Immune Mediated Inflammatory disease (UC, CD, RA, and Psoriasis) Compared to Patients without Immune Mediated Inflammatory disease.

**Disease Characteristics**	**UC** ^**a**^			**CD** ^**a**^			**RA** ^**a**^			**Psoriasis**		
	Cases	Controls	P value	Cases	Controls	P value	Cases	Controls	P value	Cases	Controls	P value
N (Weighted N)	56	150		50	132		73	175		74	193	
Percent Death	16.1	5.1	<0.001	2.3	3.9	0.003	1.3	9.5	<0.001	0.0	4.8	<0.001
Febrile Neutropenia	32.6	34.8	0.6	30.8	34.7	0.2	32.5	38.9	0.01	38.4	35.9	0.9
Bacteremia	31.4	21.0	<0.001	22.9	24.8	0.5	7.3	20.7	<0.001	12.3	17.2	0.06
ODI^a^	42.4	31.8	<0.001	39.4	36.5	0.2	16.4	36.3	<0.001	26.9	31.2	0.07
OI’s^a^	27.0	14.1	0.03	16.7	12.6	0.4	8.2	16.0	0.15	9.2	11.1	0.7
GVHD^a^	8.5	4.8	0.002	0.0	4.5	<0.001	1.5	1.7	0.7	3.7	4.6	0.4
Stomatitis	38.5	46.7	0.006	47.9	56.8	0.004	40.8	45.4	0.06	47.1	41.7	0.04
TPN^a^	24.6	20.4	0.03	16.9	15.3	0.8	8.6	9.5	0.6	9.4	8.6	0.6
Intubation	12.1	5.2	<0.001	4.1	4.4	0.5	0.0	9.6	<0.001	0.0	3.8	<0.001
Length of Stay (Days)	34.4	24.7	0.007	25.2	25.8	0.8	21.4	22.4	0.6	22.4	21.9	0.8
Total Charges ($)	354,585	226,883	0.01	226,037	252,586	0.4	180,561	212,551	0.3	197,802	203,516	0.8

^a^Abbreviations: CCI, Charlson Co-morbidity Index; TBI, Total Body Irradiation; ODI, Other Documented Infections; OI’s, Opportunistic infections; GVHD, Graft Vs. Host Disease; TPN, Total Parenteral Nutrition; UC, Ulcerative Colitis, CD, Crohn’s Disease; RA, Rheumatoid Arthritis; HSCT, Hematopoietic Stem Cell Transplantation.

**Table 4:** Multivariate Predictors of In-hospital Mortality in Patients Receiving HSCT^a^ (n = 36,018, weighted n = 181,428).

Variable	Odds Ratio (95% confidence interval)	P value
Ulcerative Colitis	3.52 (1.63–7.61)	0.001
Crohn’s Disease	0.61 (0.15–2.5)	0.5
Rheumatoid Arthritis	0.23 (0.05–0.93)	0.04
Psoriasis	0.12 (0.0–0.51)	0.006
SLE^b^	1.15 (0.49–2.69)	0.7
Diabetes Mellitus Type I	1.1 (0.58–2.12)	0.8
Multiple Sclerosis	0.99 (0.30–3.32)	0.9
Age	1.01 (1.0–1.01)	0.03
Female Gender	1.0 (0.91–1.11)	0.9
Deayo Modification of CCI^b^	1.38 (1.32–1.44)	<0.001
HSCT Type (allogeneic vs. autologous)	5.43 (4.32–6.82)	<0.001
TBI^b^	0.97 (0.84–1.12)	0.7
**Stem Cell Source**		
Peripheral Blood	Referent	
Bone Marrow	1.43 (0.33–6.21)	0.64
Cord Blood	2.2 (0.3–16.45)	0.44
Calendar Year^c^	0.91 (0.89–0.93)	<0.001

^a^Above model was also adjusted for interaction between source of stem cell and calendar year. The interaction term was statistically insignificant (p = 0.37). If by adding the interaction term, the main effect did not change more than 10%, it was removed from the final model. Effect of hospital region, teaching status, location and bed size were insignificant. So, they were removed from the final model.

^b^Abbreviations: CCI, Charlson Co-morbidity Index; TBI, Total Body Radiation; SLE, Systemic Lupus Erythematosus; HSCT, Hematopoietic Stem Cell Transplantation.

^c^Calendar year was calculated as years since 1957 (year of first HSCT) - e.g. year 2011 was treated as year 54.

### Outcomes of HSCT in UC

The UC cohort consisted of 78 patients, out of which 37 underwent allogeneic HSCT and 41 underwent autologous HSCT (Tables 1–3). Baseline characteristics are detailed in Tables 1 and 2 and the Supplementary Table S1. In multivariate analysis, UC was associated with increased mortality (odds ratio, 3.52; 95% confidence interval, 1.63–7.61; P = 0.001) (Table 4), which was consistent with the more stringent matched pair analysis (Table 3). Patients with UC who also had primary sclerosing cholangitis (PSC; n = 20, weighted n = 95) and underwent HSCT had dramatically increased mortality with an OR of 7.61 (95% CI 3.19–18.16, P < 0.001). Even after removing UC patients with PSC from the study, however, UC continued to be a predictor of increased mortality (OR 3.3; 95% CI 1.57–7.51, P = 0.001). NIS does not record the cause of mortality but in our secondary analysis we found that UC patients had significantly higher rates of bacteremia (32% vs. 19.2%, P = 0.005), other documented infections (ODIs) (46.9% vs. 32.9%, P = 0.008), opportunistic infections (OI’s) (30.8% vs. 13.4%, P < 0.001), GVHD (17.0% vs. 7.7%, P < 0.001) and intubation (12.4% vs. 4.2%, P = 0.002), as compared to patients without IMIDs (Table 1), which was consistent with results in matched pair analysis (Table 3). Among OI’s, tuberculosis (1.2% vs. 0.03%, P < 0.001) and cytomegalovirus (CMV) infections (12.8% vs. 2.9%, P < 0.001) were more common in UC patients compared to patients without IMIDs. Moreover, UC patients who had OI’s or bacteremia had dramatically higher mortality (33.3% vs. 0%, P < 0.001) compared to UC patients without OI’s and bacteremia. Thus the increase in mortality in UC patients undergoing HSCT could be secondary to increase in OI’s (tuberculosis, cytomegalovirus[CMV]) and bacteremia. UC was also associated with longer LOS in both multivariate (+4.1 days, P = 0.01) and matched pair analyses (34.4 days vs. 24.7 days, P < 0.001) (Tables 3 and 5). In addition, HSCT patients with co-morbid UC had significantly higher total charges for hospitalization in both multivariate (+$53,224, P = 0.03) and matched pair analyses ($354,585 vs. $226,883, P < 0.001) (Tables 3 and 5), which is likely related to increased complications, OIs and longer length of stay.

**Table 5:** Multivariate Predictors of Length of Stay and Total Charges for Hospitalization in Patients Receiving HSCT.

Variables	Model for Length of Stay (n = 35,484; weighted n = 179,096)		Model for Total Charges (n = 34,945; weighted n = 176,411)	
	Length of Hospitalization	P value	Total Charges	P value
Intercept	+31.9	<0.001	−162,364	0.01
Ulcerative Colitis	+4.1	0.01	+53,224	0.03
Crohn’s Disease	−0.7	0.63	−6,243	0.72
Rheumatoid Arthritis	−0.4	0.67	−13,334	0.23
Psoriasis	+0.5	0.62	−5,089	0.64
SLE^a^	+0.2	0.87	+49,002	0.02
Diabetes Mellitus Type I	+0.8	0.61	+3,580	0.80
Multiple Sclerosis	−1.7	0.14	−9,032	0.54
Age	−0.1	<0.001	−987	<0.001
Deyo Modification of CCI^a^	+0.1	0.75	+3,903	0.02
Female Gender	+0.5	0.01	+447	0.81
HSCT Type (allogeneic vs. autologous)	+9.1	<0.001	+115,049	<0.001
TBI^a^	+1.1	0.16	+4,707	0.69
**Stem Cell Source**				
Peripheral Blood	Referent			
Bone Marrow	+2.3	<0.001	+13,348	0.08
Cord Blood	+13.8	<0.001	+163,098	<0.001
Calendar Year^b^	−0.2	<0.001	+6,894	<0.001
Bacteremia	+7.6	<0.001	+73,192	<0.001
ODI^a^	+5.7	<0.001	+52,637	<0.001
TPN^a^	+4.2	<0.001	+47,876	<0.001
GVHD^a^	+12.9	<0.001	+170,016	<0.001
Intubation	+13.9	<0.001	+235,725	<0.001
**Primary Payer**				
Medicare or Medicaid	Referent		Referent	
Private Insurance	−2.1	<0.001	−12,763	<0.001
Other	−2.3	<0.001	−26,610	0.001
Urban Location of Hospital	+0.2	0.94	+37,283	<0.001
Teaching Hospital Status	+4.7	0.08	+9,453	0.72
**Hospital Region**				
Northeast	Referent		Referent	
Midwest	0.0	0.97	−20,306	0.23
South	−0.2	0.85	−28,060	0.15
West	+0.9	0.39	+21,428	0.41
**Hospital Bed Size**				
Small	Referent		Referent	
Medium	+1.4	0.49	−20,093	0.51
Large	−1.8	0.20	−48,645	0.05

^a^Abbreviations: CCI, Charlson Co-morbidity Indexradiation (exact match), gender (exact match), CCI (exact m; TBI, Total Body Radiation; SLE, Systemic Lupus Erythematosus; HSCT, Hematopoietic Stem Cell Transplantation; GVHD - Graft Vs. Host Disease; ODI, Other Documented Infections.

^b^Calendar year was calculated as years since 1957 (year of first HSCT) - e.g. year 2011 was treated as year 54.

In a subgroup analysis consisting of patients undergoing allogeneic HSCT, patients with co-morbid UC were found to have higher mortality as compared to patients without IMID (24.1% vs. 10.7%, P = 0.022). Furthermore, risk of OI’s (43.6% vs. 20.2%, P < 0.001), GVHD (29.6% vs. 17.2%, P = 0.002) and bacteremia (40.3% vs. 23.6%, P = 0.02) continued to be higher in UC patients in the allogeneic HSCT group compared to patients without IMIDs who received allogeneic HSCT (Table 2). In allogeneic HSCT, risk of tuberculosis (2.6% vs. 0.04%, *P* < 0.001) and CMV (24.8% vs. 6.0%, P < 0.001) was higher in the UC group. There was a numerical trend for increase in rates of ODI (55.9% vs. 41.0%, P = 0.06) in UC patients who underwent allogeneic HSCT. Length of stay (LOS) and total charges were higher as compared to no IMIDs.

### Outcomes of HSCT in CD

The CD cohort consisted of 72 patients, out of which 37 underwent allogeneic HSCT and 34 underwent autologous HSCT (Tables 1 and 2). Baseline characteristics are detailed in Tables 1, 2 and Supplementary Table S1. In multivariate analysis, CD was not associated with increased mortality, LOS, or total charges for hospitalization, which was consistent with the more stringent matched pair analysis (Tables 1, 3 and 5). Patients with Crohn’s disease (CD) had significantly lower rates of graft-vs-host disease (GVHD) as compared to patients without IMIDs (0% vs. 7.7%, P < 0.001), which was consistent with the matched pair analysis (Tables 1 and 3). The lower rates of GVHD may be due to the fact that our mean LOS was close to 23 days and we did not have long term follow-up data to account for patients developing GVHD after hospitalization. The rates of bacteremia, opportunistic infections, other documented infections (ODI’s), and intubation were similar between patients receiving HSCT with and without CD. Furthermore, there was no significant difference in mortality.

In our subgroup analysis consisting of patients undergoing allogeneic HSCT, patients with co-morbid CD undergoing allogeneic HSCT had no difference in mortality as compared to patients with no IMID (6.5% vs. 10.7%, P = 0.425) (Table 2). Similarly, no difference was noted in bacteremia (16.7 vs. 23.6, P = 0.30), ODI (31.0% vs. 41.00%, P = 0.27), OIs (8.7% vs. 20.2%, P = 0.16) or FN (24.1 vs 29.4, P = 0.45) as well. There was no incidence of GVHD in the CD group vs 17.2% in the no IMIDs group (P < 0.001).

### Outcomes of HSCT in RA

The RA cohort consisted of 137 patients, out of which 46 underwent allogeneic HSCT and 91 underwent autologous HSCT (Tables 1 and 2). Baseline characteristics are detailed in Tables 1, 2 and Supplementary Table S1. Coexisting RA was associated with reduced mortality (odds ratio, 0.23; 95% confidence interval, 0.05–0.93; P = 0.04) (Table 4), which was consistent with the more stringent matched pair analysis (Table 3). RA patients had significantly lower rates of bacteremia (11.6% vs. 19.2%, P = 0.04) and ODIs (22.3% vs. 32.9%, P = 0.028) as compared to patients without IMIDs (Table 1), which was also consistent in matched pair analysis (Table 3). In unmatched analyses, RA patients had significantly lower rates of OI’s (6.5% vs. 13.4%, P = 0.01), and in matched analyses, there was a numerical trend for lower OI’s (8.2% vs. 16.0%, P = 0.15). There was no significant difference in rates of LOS, total charges or GVHD in patients with RA vs. patients without IMIDs in both multivariate and matched pair analyses (Tables 3 and 5).

In our subgroup analysis consisting of patients undergoing allogeneic HSCT, patients with co-morbid RA had a trend for decrease in mortality as compared to patients with no IMIDs (4.0% vs. 10.7%, P = 0.13, although it was not statistically significant). This is likely because of a smaller number of patients in the allogeneic group. Patients with co-morbid RA that underwent allogeneic HSCT had lower incidence of ODI (25.7% vs. 41.0%, P = 0.04) and GVHD (10.7% vs. 17.2%, P = 0.04), however, no significant difference was noted in terms of other complications like bacteremia (10.9% vs. 23.6%, P = 0.06), OI (10.9% vs. 23.6%, P = 0.16) and febrile neutropenia (FN) (33.6% vs. 29.4%, P = 0.52) (Table 2). Length of stay and total charges were lower as compared to no IMIDs.

### Outcomes of HSCT in Psoriasis

The psoriasis cohort consisted of 99 patients, out of which 37 underwent allogeneic HSCT and 62 underwent autologous HSCT. Baseline characteristics are detailed in Tables 1, 2 and Supplementary Table S1. Psoriasis was associated with reduced mortality (odds ratio, 0.12; 95% confidence interval, 0.0–0.51; P = 0.006) which was consistent with the more stringent matched-pair analysis (Tables 3 and 4). In multivariate analysis, psoriasis patients had significantly lower rates of bacteremia (10.2% vs. 19.2%, P = 0.009) and intubation (0% vs. 4.2%, P < 0.001) as compared to patients without IMIDs (Table 1), which was consistent with the matched pair analysis (Table 3). However, there was no difference in rates of OI’s or GVHD in patients with psoriasis vs. patients without psoriasis, nor was psoriasis associated with increased LOS or total charges (Tables 3 and 5).

In our subgroup analysis consisting of patients undergoing allogeneic HSCT, patients with coexisting psoriasis were found to have significantly lower rates of mortality (0% vs 10.7%, P < 0.001) and bacteremia (5.1% vs 23.6%, P = 0.002) similar to the combined group (Table 2). However, other complications like ODI (40.5% vs 41.0%, P = 0.94), OI (21.1% vs 20.2%, P = 0.89), GVHD (20.3% vs. 17.2%, P = 0.37) and FN (41.0% vs 29.4%, P = 0.11) were not significantly different amongst the two groups. Length of stay and total charges were lower in patients with coexisting psoriasis as compared to no IMIDs.

### Outcomes of HSCT in SLE, DM I and MS

The outcomes of HSCT in SLE, DM I and MS subgroups as well as other predictors of outcomes from multivariate analysis are described in the Supplementary information.

## Discussion

Our study examined the impact of co-morbid IMIDs on inpatient outcomes including mortality, complications, LOS, and total charges of hospitalization in patients undergoing HSCT. A novel finding from this study is that patients with UC are at a higher risk for inpatient peri-HSCT mortality and other major complications. Furthermore, PSC, which affects approximately five percent of patients with UC, may dramatically increase the odds of HSCT mortality in patients with underlying UC^30^. The increase in mortality in UC is likely secondary to increased risk of OI, bacteremia, ODIs and GVHD. Conversely, our analysis showed a lower mortality in RA and psoriasis patients and this was associated with decreased risk of bacteremia in psoriasis and RA. There was no difference in mortality in the CD, DM, MS and SLE groups that underwent HSCT compared to patients without IMIDs. Our study shows that the difference in mortality in IMID patients undergoing HSCT is likely secondary to differences in the risk of OIs, bacteremia, and GVHD. This is in agreement with previous studies which have shown that OI, bacteremia and GVHD are independent risk factors for mortality in HSCT^31–37^. Furthermore, we identified CCI, HSCT type (allogeneic vs autologous) and calendar year as predictors of inpatient mortality. A subgroup analysis of outcomes in IMID patients who underwent allogeneic HSCT was similar to the entire study and showed that UC patients had higher mortality, psoriasis patients had lower mortality, and there was no difference in mortality in the CD, DM, MS and SLE groups. However, patients with underlying RA that underwent allogeneic HSCT showed a numerical trend for decreased mortality after HSCT, it was not statistically significant and may likely be due to the comparatively smaller sample size in the allogeneic group.

Recent studies have shown that immune mediated diseases, especially IBD and rheumatological diseases, are an important co-morbidity and increase the risk of NRM and acute GVHD in patients undergoing allogeneic HSCT^8,20,38,39^. However, in these studies, multiple IMIDs have been integrated/combined under one category and sample size of patients with comorbid IMID is modest. Our study, which evaluates the outcome of allogeneic HSCT in patients with IMID, without combining them together in one category, shows that only patients with underlying UC have worse outcomes. CD, RA, SLE, DM type I and MS had no statistically significant effect on mortality, and in fact, psoriasis patients undergoing allogeneic HSCT had better outcomes as compared to controls without IMIDs. In patients with RA who underwent allogeneic HSCT, mortality was not statistically significant, but showed a trend for lower mortality rate as compared to the no IMID group. Even though UC and CD are classified as IBD, their pathogenesis and natural history are different and their impact on the outcomes of allogeneic HSCT may differ^40^. Similarly, the impact of other IMIDs on the outcomes of HSCT can differ. Our study suggests that generalizing all IMIDs to have similar impact on outcomes of HSCT is not appropriate, and rather the risk (or lack thereof) should be determined individually for each IMID. We believe that the effect of individual IMID on HSCT outcomes needs further study using a larger and more comprehensive database like the Center for International Blood and Marrow Transplant Research (CIBMTR) registry. Established in 2004, CIBMTR operates a combined research program of the National Marrow Donor Program and the Medical College of Wisconsin^41^ and collects clinical data from allogeneic and autologous transplants performed worldwide. This registry includes comprehensive baseline donor data, baseline recipient data, and short term and long term outcome data for nearly all allogeneic transplants and approximately 80% of autologous transplants performed in the United States^41^.

UC patients who underwent HSCT were found to have a higher risk of tuberculosis (TB) and cytomegalovirus (CMV) infection. Post-HSCT, prophylactic measures are taken to prevent infection as the patients tend to be severely immunocompromised^42^. These practices often include prophylactic antibiotics, antiviral, antifungal, immune globulin infusions and vaccinations^42^. However, patients who undergo HSCT, do not routinely get anti-CMV prophylaxis unless the recipient is CMV-seropositive or if a CMV-seronegative recipient receives transplant from a seropositive donor. As per recommendations from American Society for Bone Marrow Transplantation, this high risk patient group is a candidate for preemptive anti-CMV treatment with either ganciclovir, high-dosed acyclovir or valacyclovir^42^. Our data shows increased mortality in UC patients undergoing HSCT is associated with increased risk of CMV infection, and therefore, we suggest increased vigilance for CMV in UC patients. UC patients were also found to have a higher risk of tuberculosis. Current guidelines do not recommend screening all HSCT recipients with tuberculin skin test (TST) or interferon-gamma release assay (IGRA) tests^42^. However, patients with prior active TB, prior exposure to active tuberculosis or history of positive TST or interferon-gamma release assay IGRA tests are recommended to be evaluated for active tuberculosis. UC patients who are treated with biologics like anti-tumor necrosis factor alpha (anti-TNFs) are at increased risk of TB activation and hence are screened for latent TB prior to starting therapy. As we do not have the underlying medication data we cannot assess whether UC patients that developed TB after HSCT were on concomitant biologics. However, patients with other IMIDs like CD, RA and psoriasis are also routinely treated with biologics like anti-TNFs and we did not see an increase in TB in these patients suggesting that increased risk is likely from underlying UC rather than biologic medications. Therefore, patients with UC undergoing HSCT should be vigilantly screened for risk factors and symptoms of TB. UC patients with PSC had higher mortality after HSCT. Given the small number of UC patients with PSC (n = 20), our results should be interpreted with caution, but our study does highlight the need for increased vigilance in these patients.

The main drawback of our study is that the patients who underwent HSCT were not a randomized group of patients from the diseased or healthy population. Thus, we cannot exclude the possibility that IMID patients who underwent HSCT were different from those who did not. The NIS database does not contain information on baseline donor data or long term follow-up data for the patients undergoing HSCT after discharge, hence mortality and complications post-hospital discharge could not be assessed. Another limitation of our study is that we are unable to identify the severity and activity status of underlying disease. However, by incorporating a large number of cases and stringent matching with controls with identical CCI, we mitigate the effects of disease activity. The main strength of our study is that the data are derived from the NIS, which is the largest all-payer database in the US and is representative of hospitalized patients across the country^43^. We believe that the large size and breadth of this database across the US mitigates the drawbacks outlined previously. NIS database and a similar strategy has also been successfully used to assess the safety of HSCT in other diseases like HIV^44^. Although miscoding of major cancer diagnosis and HSCT can alter the outcomes of our study, usually HSCT procedures tend to be coded well because admissions for such procedures are reimbursed maximally only when they are coded properly. In addition, we performed a stringent matching methodology of patients with IMID with up to 3 controls based on multiple variables, including indication for HSCT (exact match), type of HSCT (allogeneic vs. autologous, exact match), total body radiation (exact match), gender (exact match), CCI (exact match), age (±5 years), and calendar year (±2 years), thereby enhancing the comparability of these groups. The use of this strict matching methodology of cases helped to control for major confounders of outcomes in the study. Use of matching and regression analysis augments the validity of the findings^45^.

In summary, we found that UC was associated with increased inpatient mortality in patients undergoing HSCT. In contrast, RA and psoriasis were associated with lower inpatient mortality. There was no difference in mortality in the CD, DM, MS and SLE groups that underwent HSCT compared to patients without IMIDs. The currently used HCT-CI index that integrates RA, SLE and many IMIDs together under rheumatological diseases, and UC and CD under IBD, may need to account for IMIDs separately to predict outcomes in allogeneic HSCTs. Furthermore, UC patients had increased mortality if they had PSC and had higher risk of TB and CMV suggesting the need for increased vigilance in this cohort. Future research on long term outcomes using registries like CIBMTR and prospective studies in patients with co-morbid IMIDs undergoing HSCT, especially those with UC, are needed to confirm our findings and identify factors that can be modulated to improve and predict outcomes.

## Methods

### Data Source

The Nationwide Inpatient Sample (NIS) is the largest all-payer database of hospitalizations in the United States and was developed by the Agency of Healthcare Research and Quality and Healthcare Cost and Utilization Project (HCUP). We performed a secondary analysis of the NIS database which is publicly available and de-identifies patients. Given that this study uses data anonymized by Agency for Healthcare Research and Quality, it is exempt from Institutional Review Board (IRB) review. We have successfully used the NIS database to assess the safety of HSCT in HIV patients^44^. More details about NIS are mentioned in the supplementary information.

### Study Design and Patients

The data analysis for this cross-sectional study was prospectively planned. We used the “strengthening the reporting of observational studies in epidemiology (STROBE)” guidelines for reporting the study^46^. More details of the study design are described in the supplementary information.

### Primary and Secondary Outcomes

The primary outcome for our study was in-hospital mortality in patients undergoing HSCT with and without co-morbid IMID. The secondary outcomes included in-hospital complications of HSCT (i.e. GVHD, bacteremia, opportunistic infections (OI’s)- listed below, other documented infections, stomatitis, and febrile neutropenia), need for intubation and total parenteral nutrition, LOS, and total charges for hospitalization. These outcomes were calculated for all the patients included in the study and subsequently subgroup analysis for allogeneic HSCT groups was also performed.

The OI’s we investigated included bacterial (tuberculosis, nocardiosis, clostridium difficile, pneumococcal, legionellosis, listeriosis, nontuberculous mycobacteria), viral (cytomegalovirus, invasive herpes simplex virus), parasitic (toxoplasmosis), and fungal (pneumocystis jiroveci, invasive fungal infections (IFI)). The presence of complications was identified from primary and secondary discharge diagnoses using ICD9-CM codes (Supplementary Table S2).

### Statistical Analysis

Patients undergoing HSCT were stratified by presence or absence of IMID. Baseline characteristics were compared by Chi-square test or Fisher’s test and by ANOVA or Kruskal Wallis test for categorical and continuous variables, respectively. A P-value less than 0.05 was considered statistically significant. Multivariate logistic regression analysis was performed to identify predictors of mortality in patients undergoing HSCT. The variables in the regression analysis included presence or absence of immune -mediated inflammatory diseases listed above, age, gender, CCI, type of HSCT, source of stem cell (peripheral blood, bone marrow, and cord blood), presence or absence of total body irradiation (TBI), primary payer, hospital region, urban location of hospital, teaching hospital status, hospital bed-size, and calendar year. In the regression models, the interaction term for each significant variable with calendar year was added to control for change in outcomes of HSCT with time. If by adding the interaction term, the main effect did not change more than 10%, it was removed from the final model. Regression analyses also took into account the stratified two-stage cluster design of the NIS using survey procedures in SAS, incorporating individual discharge-level weights. Multivariate linear regression analysis, including these same variables, was performed to examine association of IMID with LOS and total charges for the hospitalization. SAS version 9.2 (SAS Institute Inc, Cary, North Carolina) was utilized for all analysis. The regression analyses may not control for underlying disease for performing HSCT; and because the outcome of HSCT may depend in part on the underlying indication, we conducted a matched pair analysis to control for this in addition to other significant predictors found in multivariate regression analyses.

### Matched Pair Analysis

We used individual matching of cases (HSCT patients with a given IMID) with up to 3 controls (HSCT patients without IMID) to further determine if the difference in outcomes for patients with an IMID were independent of indication for HSCT, type of HSCT, age, gender, CCI and calendar year. We used a greedy matching algorithm^47^ to match for indication for HSCT (exact match), type of HSCT (allogeneic vs. autologous, exact match), total body radiation (exact match), gender (exact match), CCI (exact match), age (±5 years), and calendar year (±2 years). More details are mentioned in the supplementary information.

### Total charges

The total charges for each hospitalization were adjusted for inflation by using Consumer Price Index (CPI) data released by the Bureau of Labor Statistics (BLS)^48,49^. The adjusted total charges were calculated by multiplying the CPI inflation factor for the given year as compared to 2011.

## Electronic supplementary material

Supplementary information

## References

[1] Copelan EA (2006). Hematopoietic stem-cell transplantation. N Engl J Med.

[2] Marr KA, Carter RA, Crippa F, Wald A, Corey L (2002). Epidemiology and outcome of mould infections in hematopoietic stem cell transplant recipients. Clin Infect Dis.

[3] Boeckh M, Nichols WG (2004). The impact of cytomegalovirus serostatus of donor and recipient before hematopoietic stem cell transplantation in the era of antiviral prophylaxis and preemptive therapy. Blood.

[4] Gratwohl A (2005). Cause of death after allogeneic haematopoietic stem cell transplantation (HSCT) in early leukaemias: an EBMT analysis of lethal infectious complications and changes over calendar time. Bone Marrow Transplant.

[5] Gooley TA (2010). Reduced mortality after allogeneic hematopoietic-cell transplantation. N Engl J Med.

[6] Hahn T (2013). Significant improvement in survival after allogeneic hematopoietic cell transplantation during a period of significantly increased use, older recipient age, and use of unrelated donors. J Clin Oncol.

[7] McCarthy PL (2013). Trends in use of and survival after autologous hematopoietic cell transplantation in North America, 1995–2005: significant improvement in survival for lymphoma and myeloma during a period of increasing recipient age. Biol Blood Marrow Transplant.

[8] Sorror ML (2005). Hematopoietic cell transplantation (HCT)-specific comorbidity index: a new tool for risk assessment before allogeneic HCT. Blood.

[9] National Institutes of Health Autoimmune Disease Coordinating Committee Report. In. Bethesda (MD), (https://www.niaid.nih.gov/topics/autoimmune/Pages/coordComm.aspx) (2002).

[10] Walsh SJ, Rau LM (2000). Autoimmune diseases: a leading cause of death among young and middle-aged women in the United States. Am J Public Health.

[11] Cucchiara S, Stronati L (2011). Incidence in pediatric IBD is rising: help from health administrative data. Inflamm Bowel Dis.

[12] Molodecky, N. A. *et al*. Increasing incidence and prevalence of the inflammatory bowel diseases with time, based on systematic review. *Gastroenterology* **142**, 46–54 e42, quize30, 10.1053/j.gastro.2011.10.001 (2012). 10.1053/j.gastro.2011.10.001 22001864

[13] Myasoedova E, Crowson CS, Kremers HM, Therneau TM, Gabriel SE (2010). Is the incidence of rheumatoid arthritis rising?: results from Olmsted County, Minnesota, 1955–2007. Arthritis Rheum.

[14] Somers EC (2014). Population-based incidence and prevalence of systemic lupus erythematosus: the Michigan Lupus Epidemiology and Surveillance program. Arthritis Rheumatol.

[15] Majhail NS, Omondi NA, Denzen E, Murphy EA, Rizzo JD (2010). Access to hematopoietic cell transplantation in the United States. Biol Blood Marrow Transplant.

[16] Bach JF (2002). The effect of infections on susceptibility to autoimmune and allergic diseases. N Engl J Med.

[17] Cho JH, Gregersen PK (2011). Genomics and the multifactorial nature of human autoimmune disease. N Engl J Med.

[18] Dave M, Papadakis KA, Faubion WA (2014). Immunology of inflammatory bowel disease and molecular targets for biologics. Gastroenterol Clin North Am.

[19] Davidson A, Diamond B (2001). Autoimmune diseases. N Engl J Med.

[20] Sorror ML (2014). Pretransplant comorbidities predict severity of acute graft-versus-host disease and subsequent mortality. Blood.

[21] Rabian F (2016). Influence of Previous Inflammatory Bowel Disease on the Outcome of Allogeneic Hematopoietic Stem Cell Transplantation: A Matched-Pair Analysis. Biol Blood Marrow Transplant.

[22] Atkinson MA, Eisenbarth GS (2001). Type 1 diabetes: new perspectives on disease pathogenesis and treatment. Lancet.

[23] Cooles FA, Isaacs JD (2011). Pathophysiology of rheumatoid arthritis. Curr Opin Rheumatol.

[24] Emery P (2006). Treatment of rheumatoid arthritis. BMJ.

[25] Falk RJ (2000). Treatment of lupus nephritis–a work in progress. N Engl J Med.

[26] Mok CC, Lau CS (2003). Pathogenesis of systemic lupus erythematosus. J Clin Pathol.

[27] Sartor RB (2006). Mechanisms of disease: pathogenesis of Crohn’s disease and ulcerative colitis. Nat Clin Pract Gastroenterol Hepatol.

[28] Hatzimichael E, Tuthill M (2010). Hematopoietic stem cell transplantation. Stem Cells Cloning.

[29] Jones JA (2008). In-hospital complications of autologous hematopoietic stem cell transplantation for lymphoid malignancies: clinical and economic outcomes from the Nationwide Inpatient Sample. Cancer.

[30] Olsson R (1991). Prevalence of primary sclerosing cholangitis in patients with ulcerative colitis. Gastroenterology.

[31] Daikeler T, Tichelli A, Passweg J (2012). Complications of autologous hematopoietic stem cell transplantation for patients with autoimmune diseases. Pediatr Res.

[32] Inagaki J (2016). Effect of Cytomegalovirus Reactivation on Relapse after Allogeneic Hematopoietic Stem Cell Transplantation in Pediatric Acute Leukemia. Biol Blood Marrow Transplant.

[33] Koldehoff M, Beelen DW, Elmaagacli AH (2013). Increased susceptibility for aspergillosis and post-transplant immune deficiency in patients with gene variants of TLR4 after stem cell transplantation. Transpl Infect Dis.

[34] Mikulska M (2009). Blood stream infections in allogeneic hematopoietic stem cell transplant recipients: reemergence of Gram-negative rods and increasing antibiotic resistance. Biol Blood Marrow Transplant.

[35] Poutsiaka DD, Munson D, Price LL, Chan GW, Snydman DR (2011). Blood stream infection (BSI) and acute GVHD after hematopoietic SCT (HSCT) are associated. Bone Marrow Transplant.

[36] Uhlin M (2014). Risk factors for Epstein-Barr virus-related post-transplant lymphoproliferative disease after allogeneic hematopoietic stem cell transplantation. Haematologica.

[37] Wang LR, Dong LJ, Zhang MJ, Lu DP (2006). The impact of human herpesvirus 6B reactivation on early complications following allogeneic hematopoietic stem cell transplantation. Biol Blood Marrow Transplant.

[38] Barba P (2017). Hematopoietic Cell Transplantation Comorbidity Index Predicts Outcomes in Patients with Acute Myeloid Leukemia and Myelodysplastic Syndromes Receiving CD34+ Selected Grafts for Allogeneic Hematopoietic Cell Transplantation. Biol Blood Marrow Transplant.

[39] Sorror ML (2007). Hematopoietic cell transplantation specific comorbidity index as an outcome predictor for patients with acute myeloid leukemia in first remission: combined FHCRC and MDACC experiences. Blood.

[40] Cosnes J, Gower-Rousseau C, Seksik P, Cortot A (2011). Epidemiology and natural history of inflammatory bowel diseases. Gastroenterology.

[41] Center for International Blood and Marrow Transplant and Research (CIBMTR) home page. Available from: https://www.cibmtr.org/Data/Available/Pages/index.aspx.

[42] Tomblyn M (2009). Guidelines for preventing infectious complications among hematopoietic cell transplantation recipients: a global perspective. Biol Blood Marrow Transplant.

[43] HCUP Nationwide Inpatient Sample (NIS). Healthcare Cost and Utilization Project (HCUP). Edited by: Agency for Healthcare Research and Quality, Rockville, MD; 2007–2009. (http://www.hcup-us.ahrq.gov/nisoverview.jsp).

[44] Mehta, K., Im, A., Rahman, F., Wang, H. & Veldkamp, P. Epidemiology and Outcomes of Hematopoietic Stem Cell Transplant in HIV (+) Patients from 1998 to 2012: A Nationwide Analysis. *Clin Infect Dis*, 10.1093/cid/ciy010 (2018). 10.1093/cid/ciy010 29325063

[45] de Graaf MA, Jager KJ, Zoccali C, Dekker FW (2011). Matching, an appealing method to avoid confounding?. Nephron Clin Pract.

[46] von Elm E (2014). The Strengthening the Reporting of Observational Studies in Epidemiology (STROBE) Statement: guidelines for reporting observational studies. Int J Surg.

[47] Cormen TH CEL, a. R. L. R. Introduction To Algorithms: The MIT Press, (2011).

[48] CPI Inflation Calculator. Edited by: Bureau of Labor Statistics, (2015).

[49] Patel NJ (2014). Contemporary trends of hospitalization for atrial fibrillation in the United States, 2000 through 2010: implications for healthcare planning. Circulation.

