# Supplementary material for: In-patient outcomes of Hematopoietic Stem Cell Transplantation in Patients with Immune Mediated Inflammatory Diseases: A Nationwide Study
Source: Sci Rep. 2018 May 1;8:6825. doi: 10.1038/s41598-018-24060-4 (PMC5931606; doi:10.1038/s41598-018-24060-4)
Supplement: Supplementary file 1 — Supplementary information [file 41598_2018_24060_MOESM1_ESM.pdf]

Supplementary information:

In-patient outcomes of Hematopoietic Stem Cell Transplantation in Patients with Immune Mediated Inflammatory Diseases: A Nationwide Study

Kathan Mehta, MBBS, MPH [1]; Palashkumar Jaiswal, MBBS[2]; Farren Briggs, PhD, ScM [3]; William A. Faubion, MD[4]; James H. Tabibian, MD, PhD[5]; Fabio Cominelli, MD, PhD[6]; Maneesh Dave, MD, MPH[6*]

Author Affiliations:

[1]Division of Hematology and Oncology, University of Pittsburgh Medical Center, Pittsburgh, Pennsylvania, USA.
[2]Department of Internal Medicine, John H Stroger, Jr. Hospital of Cook County, Chicago, Illinois, USA
[3]Department of Epidemiology and Biostatistics, Case Western Reserve University, Cleveland, Ohio, USA.
[4]Division of Gastroenterology and Hepatology, Mayo Clinic, Rochester, Minnesota, USA.
[5]Division of Gastroenterology, Department of Medicine Olive View-UCLA Medical Center, Sylmar, CA, United States.
[6]Division of Gastroenterology and Liver Disease, University Hospitals, Case Western Reserve University, Cleveland, Ohio, USA.

***Corresponding Author:** Maneesh Dave, MD, MPH

Email: maneesh.dave@case.edu

Results:

Outcomes of HSCT in SLE, DM I and MS:

The SLE group consisted of 74 patients (26 underwent allogeneic HSCT and 47 underwent autologous HSCT), the DM group consisted of 143 patients (64 underwent allogeneic HSCT and 78 underwent autologous HSCT), and the MS group consisted of 91 patients (19 underwent allogeneic HSCT and 72 underwent autologous HSCT). Baseline characteristics are detailed in Tables 1, 2 and Supplementary Table S1. On both multivariate (Tables 1, 4 and 5) and matched pair analysis (Supplementary Table S3), patients with SLE, DM I, or MS who underwent HSCT had no difference in mortality, LOS, or total charges for hospitalization compared to patients without these IMIDs.

Similar to multivariate analyses, there was no statistical difference between the mortality rates in patients with co-morbid SLE (19.2% vs. 10.7%, P=0.09), DM I (9.9% vs. 10.7%, P=0.84) and MS (10.7% vs. 10.7%, P=0.99) who underwent allogeneic HSCT as compared to patients with no IMIDs. However, the SLE group showed a trend towards higher mortality. Similarly, no significant difference was noted in incidence of complications, namely bacteremia, ODI, OI or FN as compared to patients with no IMIDs. GVHD was significantly lower in DM I group but no significant difference was noted in the SLE or MS groups (Table 2). Length of stay and total charges were lower in the MS group as compared to the no IMIDs group, while DM and SLE had no influence on either group in patients who underwent allogeneic HSCT.

Trends of HSCT, type of HSCT, sources of stem cells, and total body irradiation in United States from 1998 to 2011 are shown in Figure S1 and Figure S2.

Other Outcomes of Interest:

In multivariate analysis, allogeneic HSCT was associated with higher mortality (OR 5.43, 95% CI 4.32 - 6.82, p<0.001), longer length of stay (LOS) (+9.1 days, p<0.001) and higher total charges (+$115,049, p<0.001) as compared to autologous HSCT (Table 4). Other significant predictors of longer LOS and higher total charges included presence of complications, namely GVHD (+12.9 days, +$170,016,

p<0.001), bacteremia (+7.6 days, +$73,192, p<0.001), ODI's (+5.7 days, +$52,637, p<0.001), total parenteral nutrition (+4.2 days, +$47,876, p<0.001), intubation (+13.9 days, +$235,725, p<0.001) and use of cord blood as a source of stem cells (+13.8 days, +$163,098, p<0.001).

Method:

Data Source:

NIS consists of a database that combines the data collection efforts of state data organizations, hospital associations, private data organizations, and the federal government to create a national information resource of patient-level health care data [1,2]. NIS is a 20% stratified sample of all US community hospitals and academic medical centers and collects data on more than 8 million hospital discharges each year. NIS database uses National Health Survey Strata to weigh each participating hospital. Discharge weights are provided for each entry and can be used to project to a nationally representative population. Each hospitalization is de-identified and contains one primary discharge diagnosis and up to 24 secondary discharge diagnoses. In addition, each discharge contains information on demographic characteristics of patients (age, race, gender, and ethnicity), characteristics of the hospital (teaching vs. non-teaching, hospital bed size, urban vs. rural location, and region), insurance status, LOS, total charges for the hospitalization, one primary procedure and up to 14 secondary procedures performed during the hospitalization, and in-hospital mortality status. The discharge diagnoses and procedures are recorded as International Classification of Diseases, 9th edition, Clinical Modification (ICD9-CM) diagnosis codes and ICD9-CM procedure codes, respectively. NIS database has been extensively used in the past to study both benign and malignant hematological diseases [3,4].

Study Design and Patients:

We queried NIS database between 1998 and 2011 for HSCT using ICD9-CM procedure code (41.0x). The IMIDs selected for this study were UC, CD, RA, SLE, DM I, MS, psoriasis and SS based on

their increased prevalence in the US and the NIS database containing at least 50 patients with each of these IMIDs. We first performed the analysis on all the patients with aforementioned IMIDs who underwent HSCT for existing malignant or non-malignant disease. Subsequently, for each IMID, we also performed separate sub-group analysis for allogeneic HSCT. We specifically excluded all the patients who received HSCT primarily for treatment of underlying IMID and those who had multiple IMIDs. The indications for HSCT and presence of co-existing IMID (Supplementary Table S4) were identified from primary and secondary discharge diagnoses using ICD9-CM codes (Supplementary Table S2).

The severity of co-morbid conditions other than IMIDs (such as heart disease or liver disease) for a given patient was quantified by determining the Deyo’s modification of Charlson co-morbidity index (CCI) as described [5]. This index is calculated by identifying the presence of 17 co-morbid conditions and assigning differential weights to each condition. The total of these weights is defined as the CCI. The index ranges from 0-33, with higher scores indicating severe co-morbid conditions, and has been used extensively in clinical research studies [6-8]. The co-morbid conditions included in the CCI were identified by using ICD9-CM codes of primary and secondary discharge diagnoses for each patient.

Matched Pair Analysis:

While matching for indication for HSCT, the highest level of sub-classification for a given disease available through ICD9 codes was used. For example, cases with multiple myeloma in remission (ICD9 - 203.01) were only matched to controls with multiple myeloma in remission (ICD9 - 203.01) and not to patients with multiple myeloma in relapse (ICD9- 203.02). SAS Macro, available on Mayo Clinic’s website, was used to implement the matching algorithm [9]; this macro has been previously used in published research to generate matched datasets [10]. A matched cohort was created separately for each IMID to examine differences in outcomes between the two groups. The differences between all the matching variables were insignificant by at least $P > 0.2$ (Supplementary Table S5). For each matched cohort, differences in outcomes were assessed by performing a Cochran-mantel-haenszel chi-square test with match-ID as a stratification variable for categorical variables and linear regression with match-ID as a stratification

variable for continuous variables. Since, Cochran-mantel-haenszel odds ratio did not change direction of association for any variable, we only reported *P* values. If, after matching, there were less than 25 cases for a given IMID that could be matched to controls, then we excluded that IMID from our study. For IBD, there were more than 50 patients each for UC and CD, and previous studies utilizing NIS have been able to study them separately with high accuracy; therefore, we analyzed CD and UC separately [7,11-13].

Supplementary Table legends:

Supplementary Table S1: Additional Baseline Characteristics in Patients Undergoing HSCT from 1998 to 2011.

Supplementary Table S2: ICD-9 CM Codes Used in the Analysis

Supplementary Table S3: Outcomes of HSCT in Matched Sample of Patients with Immune mediated Inflammatory disease (SLE, MS and DM I) Compared to Patients without Immune mediated Inflammatory disease.

Supplementary Table S4: Major Indications of HSCT in Patients with Different Co-morbid IMIDs.

Supplementary Table S5: Baseline Characteristics of Matched Sample of Patients Undergoing HSCT with Immune-mediated inflammatory diseases (Cases) Compared to Patients without immune-mediated inflammatory diseases (Control)

Supplementary Figure Legends:

Figure S1: Trend of HSCT Performed in United States from 1998 to 2011.

The un-weighted numbers represent actual patients receiving HSCT identified in the dataset. The weighted numbers represent patients receiving HSCT when projected to all US hospitalizations.

Figure S2: Trends of HSCT, Type of HSCT, Sources of Stem Cells, and Total Body Irradiation in United States from 1998 to 2011.

Trends of HSCT (panel - a), type of HSCT (panel - b), sources of stem cells (panel - c), and total body irradiation (panel - d) in United States from 1998 to 2011. Panel b, c and d only include weighted data. Abbreviations: TBI = Total body irradiation.

Supplementary Table S1: Additional Baseline Characteristics in Patients Undergoing HSCT from 1998 to 2011.

**Baseline characteristics**	**UC**	**CD**	**RA**	**Psoriasis**	**SLE**	**DM I**	**MS**	**No IMID**
Median household income category for patient's zip code (%)								
0-25th percentile	19.0	20.2	11.1	19.2	14.3	8.1	15.5	14.5
26-50th percentile	15.8	20.7	25.3	14.3	20.3	18.6	13.3	20.5
51-75th percentile	26.1	28.4	28.4	30.9	23.5	27.1	33.1	25.5
76-100th percentile	36.6	27.9	32.1	34.9	39.2	42.1	35.9	36.1
Primary payer (%)								
Medicare / Medicaid	31.9	22.7	36.6	23.3	36.1	22.1	31.6	26.5
Private including HMO	65.6	72.1	62.0	68.6	62.6	69.9	55.6	66.6
Self-pay/no charge/other	2.5	5.2	1.4	8.0	1.3	7.4	12.8	6.6
Hospital characteristics (%)								
Hospital bed size								
Small	16.4	9.1	9.4	10.8	9.7	11.5	14.6	14.7
Medium	8.8	7.4	7.1	2.1	1.2	6.5	.	9.5
Large	70.6	82.2	82.1	85.3	89.1	82.0	85.4	74.8
Hospital Location								
Urban	95.7	95.9	98.6	94.1	97.4	100.0	100.0	98.0
Hospital Region								
Northeast	28.7	32.3	23.1	35.3	10.5	24.4	12.1	22.2
Midwest or North Central	26.9	35.2	28.9	28.5	38.7	23.7	54.3	24.9
South	25.9	13.2	28.5	27.1	26.1	29.8	13.0	28.3
West	18.5	19.3	19.5	9.1	24.7	22.1	20.7	24.6
Hospital Teaching status								
Teaching	93.4	94.9	94.4	95.2	96.3	96.6	98.9	96.1
Admission types (%)								
Emergent/Urgent	23.8	21.4	18.2	12.5	21.4	21.9	31.1	19.0
Missing	10.8	7.3	9.3	3.1	20.8	13.7	10.0	14.6
Admission day (%)								
Weekend	10.2	16.7	6.2	5.8	4.0	11.0	8.0	9.6
Disposition (%)								
Home	82.2	95.1	94.4	99.0	90.4	85.0	90.3	92.2
Facility	1.4	1.6	3.5	0.0	2.8	5.7	6.4	1.8

Abbreviations: IMID, Immune mediated Inflammatory disease; HMO, Health Maintenance Organization; HSCT, Hematopoietic Stem Cell Transplantation; UC, Ulcerative Colitis; CD, Crohn's Disease; RA, Rheumatoid Arthritis; DM, Diabetes Mellitus; SLE, Systemic Lupus Erythematosus; MS, Multiple Sclerosis

Supplementary Table S1: Additional Baseline Characteristics in Patients Undergoing HSCT from 1998 to 2011.

Supplementary Table S2: ICD-9 CM Codes Used in the Analysis

**Procedures**	**ICD9-CM Code**
HSCT	41.x
Allogeneic HSCT	41.02, 41.03, 41.05, 41.06, 41.08
Autologous HSCT	41.01, 41.04, 41.07, 41.09
Peripheral blood HSCT	41.04, 41.07, 41.05, 41.08
Bone marrow HSCT	41.00, 41.01, 41.09, 41.02, 41.03
Cord blood HSCT	41.06
TBI conditioning	92.24, 92.26, 92.77, 92.29
TPN	99.15
Intubation	96.01–96.05
**Immune Mediated Inflammatory Diseases**	
Ulcerative colitis	556.x
Crohn's disease	555.x
Rheumatoid arthritis	714.0, 714.1, 714.2
Systemic lupus erythematosus	710.0
Diabetes mellitus type I	250.01, 250.03, 250.11, 250.13, 250.21, 250.23, 250.31, 250.33, 250.41, 250.43, 250.51, 250.53, 250.61, 250.63, 250.71, 250.73, 250.81, 250.83, 250.91, 250.93
Psoriasis	696.0, 696.1, 696.8
Multiple sclerosis	340
Systemic sclerosis	710.1
**Possible indications of HSCT**	
Malignant neoplasm of ovary and other uterine adnexa	183.x
Malignant neoplasm of testis	186.x
Neoplasm of uncertain behavior of endocrine glands and nervous system	237.x
Lymphosarcoma and reticulosarcoma and other specified malignant tumors of lymphatic tissue	200.x
Hodgkin's disease	201.x
Other malignant neoplasms of lymphoid and histiocytic tissue	202.x
Multiple myeloma and immunoproliferative neoplasms	203.x
Lymphoid leukemia	204.x
Myeloid leukemia	205.x
Monocytic leukemia	206.x
Other specified leukemia	207.x
Leukemia of unspecified cell type	208.x
Malignant neoplasm of bone and articular cartilage	170.x
Malignant neoplasm of connective and other soft tissue	171.x
Malignant neoplasm of brain	191.x
Malignant neoplasm of other and unspecified parts of nervous system	192.x
Aplastic anemia and other bone marrow failure syndromes	284.x
Neoplasm of uncertain behavior of other lymphatic and hematopoietic tissues	238.7x
Amyloidosis	277.3x
Thalassemias	282.4x
Sickle-cell disease	282.6x

Genetic anomalies of leukocytes	288.2
Malignant neoplasm of pineal gland	194.4
Malignant neoplasm of adrenal gland	194.0
Personal history of lymphosarcoma and reticulosarcoma	V10.71
Personal history of Hodgkin's disease	V10.72
Personal history of other lymphatic and hematopoietic neoplasms	V10.79
Personal history of malignant neoplasm of brain	V10.85
Personal history of malignant neoplasm of other parts of nervous system	V10.86
Wiskott-Aldrich syndrome	279.12
Combined immunity deficiency	279.2
Osteopetrosis	756.52
**Adverse reactions of HSCT**	
Febrile neutropenia	288.0
Bacteremia	38.xx, 790.7
Other documented infection	001.xx-037.xx, 039.xx-135.xx, 487.1, 487.8, 490, 465.xx., 466.xx, 595.0, 595.9, 595.89, 681.xx-682.xx
Opportunistic infections	078.5, 484.1,031.x, 130.x, 136.3, 01x.x, 116.x, 118.x, 117.3, 112.4, 112.5, 114.0, 114.2, 114.3, 117.5, 117.6, 117.9, 117.7, 112.81, 112.83, 112.84, 112.85, 115.01, 115.02, 115.03, 115.04, 115.05, 115.11, 115.12, 115.13, 115.14, 115.15, 115.91, 115.92, 115.93, 115.94, 115.95, 003.1, 046.3, 054.3, 054.5, 008.45, 078.1, 079.4, 487.x, 039.9, 320.1, 567.1 , 038.2, 041.2, 481.x, 482.84, 027.0, 127.2
Stomatitis	528.x
GVHD	279.5

Abbreviations: ICD-9 CM, International Classification of Diseases, 9th Revision, Clinical Modification; HSCT, Hematopoietic Stem Cell Transplantation; TBI, Total Body Radiation; GVHD, Graft vs Host disease; TPN, Total peripheral nutrition.

Supplementary Table S2: ICD-9 CM Codes Used in the Analysis

Supplementary Table S3: Outcomes of HSCT in Matched Sample of Patients with Immune mediated Inflammatory diseases (SLE, MS and DM I) Compared to Patients without Immune mediated Inflammatory disease.

**Disease Characteristics**	**SLE**			**MS**			**Type I DM**		
	Cases	Controls	P value	Cases	Controls	P value	Cases	Controls	P value
N (Weighted N)	28 (145)	64 (315)		36 (181)	92 (464)		72 (354)	178 (904)	
Percent Death	6.6	10.6	<0.001	8.3	5.2	0.1	8.8	8.4	0.8
Febrile Neutropenia	39.6	45.3	0.7	30.6	32.3	0.8	22.9	33.0	0.0009
Bacteremia	15.1	16.9	0.002	5.4	9.7	0.06	22.0	18.1	0.4
ODI	28.1	28.3	0.06	41.7	40.8	0.6	36.3	32.5	0.3
OI's	10.3	12.2	0.8	27.5	18.3	0.3	9.9	16.3	0.2
GVHD	0.0	1.6	0.1	3.1	4.1	0.8	1.3	2.2	0.3
Stomatitis	32.6	38.2	1.0	41.4	44.6	0.7	39.9	40.8	0.8
TPN	3.5	13.9	0.0003	4.1	16.0	<0.0001	21.4	16.0	0.02
Intubation	3.5	10.5	<0.0001	4.9	3.2	0.3	4.4	7.0	0.04
Length of Stay (Days)	25.5	26.6	0.8	25.3	26.4	0.6	26.4	25.9	0.8
Total Charges ($)	265,948	208,975	0.3	196,721	237,273	0.1	207,855	225,460	0.5

Abbreviations: CCI, Charlson Co-morbidity Index; DM, Diabetes Mellitus; HSCT, Hematopoietic Stem Cell Transplant; MS, Multiple Sclerosis; TBI, Total Body Irradiation; ODI, Other Documented Infections; OI's, Opportunistic infections; GVHD, Graft Vs. Host Disease; SLE, Systemic Lupus Erythematosus; TPN, Total Parenteral Nutrition.

Supplementary Table S3: Outcomes of HSCT in Matched Sample of Patients with Immune mediated Inflammatory diseases (SLE, MS and DM I) Compared to Patients without Immune mediated Inflammatory disease.

Supplementary Table S4: Major Indications of HSCT in Patients with Different Co-morbid IMIDs

Indications	UC	CD	RA	Psoriasis	SLE	DM1	MS	No IMID
**Indications of Allogenic HSCT (%)**								
HL	2.91	0.00	7.43	5.05	0.00	0.00	0.00	2.91
NHL	16.61	14.37	16.08	16.91	7.99	8.24	10.43	13.93
Leukemia	61.22	50.98	47.81	64.84	53.94	61.75	64.93	62.69
MM	0.00	5.61	0.00	2.57	0.00	6.36	4.72	3.41
**Indications of Autologous HSCT (%)**								
HL	5.36	0.00	8.06	15.19	6.33	14.09	0.00	10.14
NHL	29.98	29.52	42.11	42.36	23.19	27.82	8.61	26.32
Leukemia	5.09	18.15	1.16	2.01	4.53	8.90	2.63	6.15
MM	46.05	32.58	38.01	34.32	26.06	31.38	8.58	35.74

Abbreviations: CD, Crohn's Disease; DM, Diabetes Mellitus; HL, Hodgkin's Lymphoma; HSCT, Hematopoietic Stem Cell Transplant; IMID, Immune Mediated Inflammatory Disease; MM, Multiple Myeloma; MS, Multiple Sclerosis; NHL, Non-Hodgkin's Lymphoma; RA, Rheumatoid Arthritis; UC, Ulcerative Colitis; SLE, Systemic Lupus Erythematosus.

Supplementary Table S4: Major Indications of HSCT in Patients with Different Co-morbid IMIDs

Supplementary Table S5: Baseline Characteristics of Matched Sample of Patients Undergoing HSCT with Immune-mediated inflammatory diseases (Cases) Compared to Patients without immune-mediated inflammatory diseases (Control) [a]

Disease Characteristics	UC		CD		RA		SLE		MS		Psoriasis		DM I	
	Cases	Controls	Cases	Controls	Cases	Controls	Cases	Controls	Cases	Controls	Cases	Controls	Cases	Controls
N (Weighted N)	56 (281)	150 (754)	50 (253)	132 (657)	73 (355)	175 (878)	28 (145)	64 (315)	36 (181)	92 (464)	74 (372)	193 (976)	72 (354)	178 (904)
Age	48.9	48.3	52.7	52.9	58.3	57.9	51.6	54.7	49.6	50.8	52.3	52.3	50.5	50.4
Male	57.0	57.3	53.5	55.3	40.7	42.8	11.8	11.7	23.9	26.6	71.5	71.4	55.6	53.6
Charlson Score	2.04	2.11	1.81	1.76	3.08	3.08	2.88	3.06	1.81	1.86	2.13	2.11	3.02	2.98
Type of HSCT														
Allogenic	44.8	44.4	52.9	54.8	28.4	27.2	35.4	29.2	45.4	45.2	37.9	38.6	37.5	33.8
Autologous	55.2	55.6	47.1	45.3	71.6	72.8	64.6	70.8	54.6	54.8	62.1	61.4	62.5	66.2
Conditioning														
Total Body Irradiation (TBI)	9.2	8.9	14.3	13.6	5.7	3.5	3.6	1.4	15.5	11.9	6.8	6.9	8.6	6.7
No TBI	90.8	91.1	85.7	86.5	94.3	96.5	96.4	98.6	84.5	88.1	93.2	93.1	91.4	93.3
Calendar Year (Median)	2007	2007	2008	2007	2008	2008	2005	2007	2007	2008	2008	2008	2003	2004

a. All between the group P values were insignificant by at least p>0.2.

Abbreviations: HMO, Health Maintenance Organization ; HSCT, Hematopoietic Stem Cell Transplantation; UC, Ulcerative Colitis; CD, Crohn's Disease; RA, Rheumatoid Arthritis; DM, Diabetes Mellitus; SLE, Systemic Lupus Erythematosus; MS, Multiple Sclerosis.

Supplementary Table S5: Baseline Characteristics of Matched Sample of Patients Undergoing HSCT with Immune-mediated inflammatory diseases (Cases) Compared to Patients without immune-mediated inflammatory diseases (Control)

Supplementary Figures:

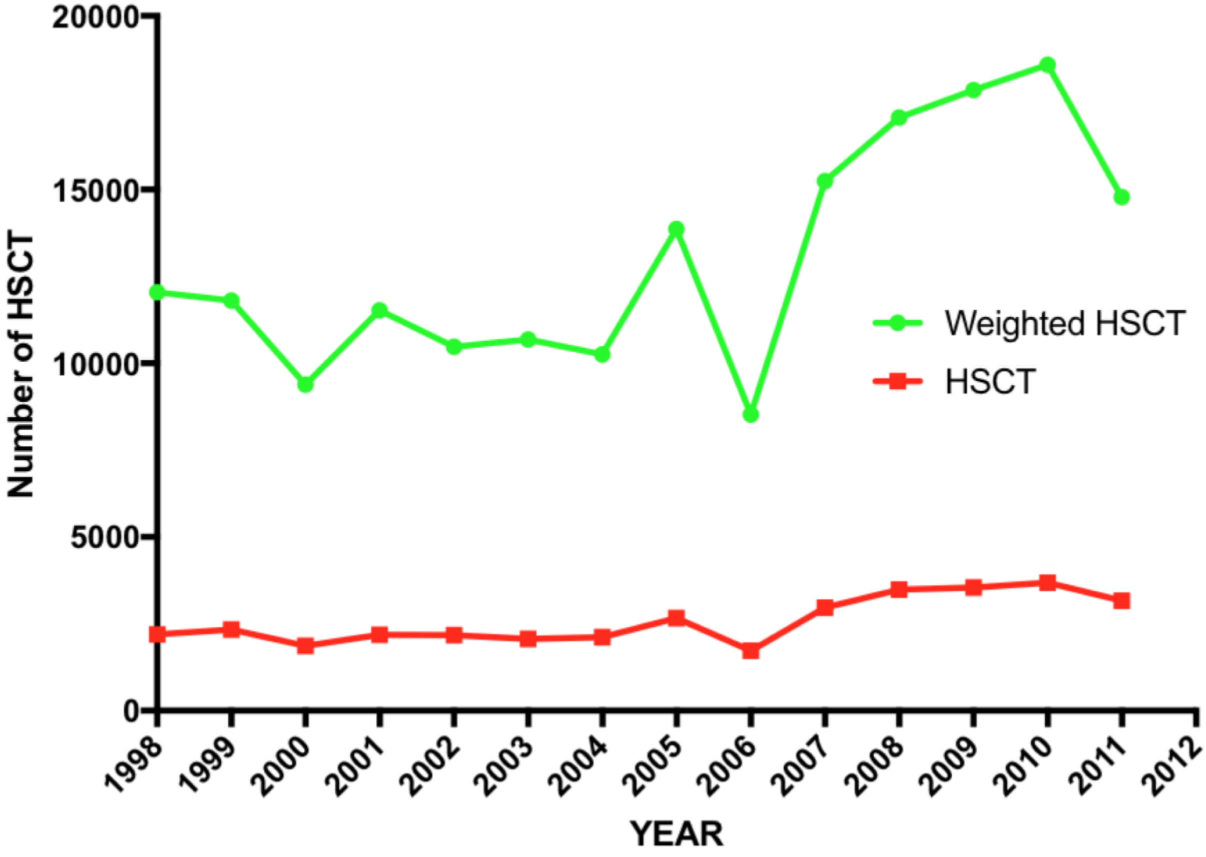

Figure S1: Trend of HSCT Performed in United States from 1998 to 2011. The un-weighted numbers represent actual patients receiving HSCT identified in the dataset. The weighted numbers represent patients receiving HSCT when projected to all US hospitalizations.

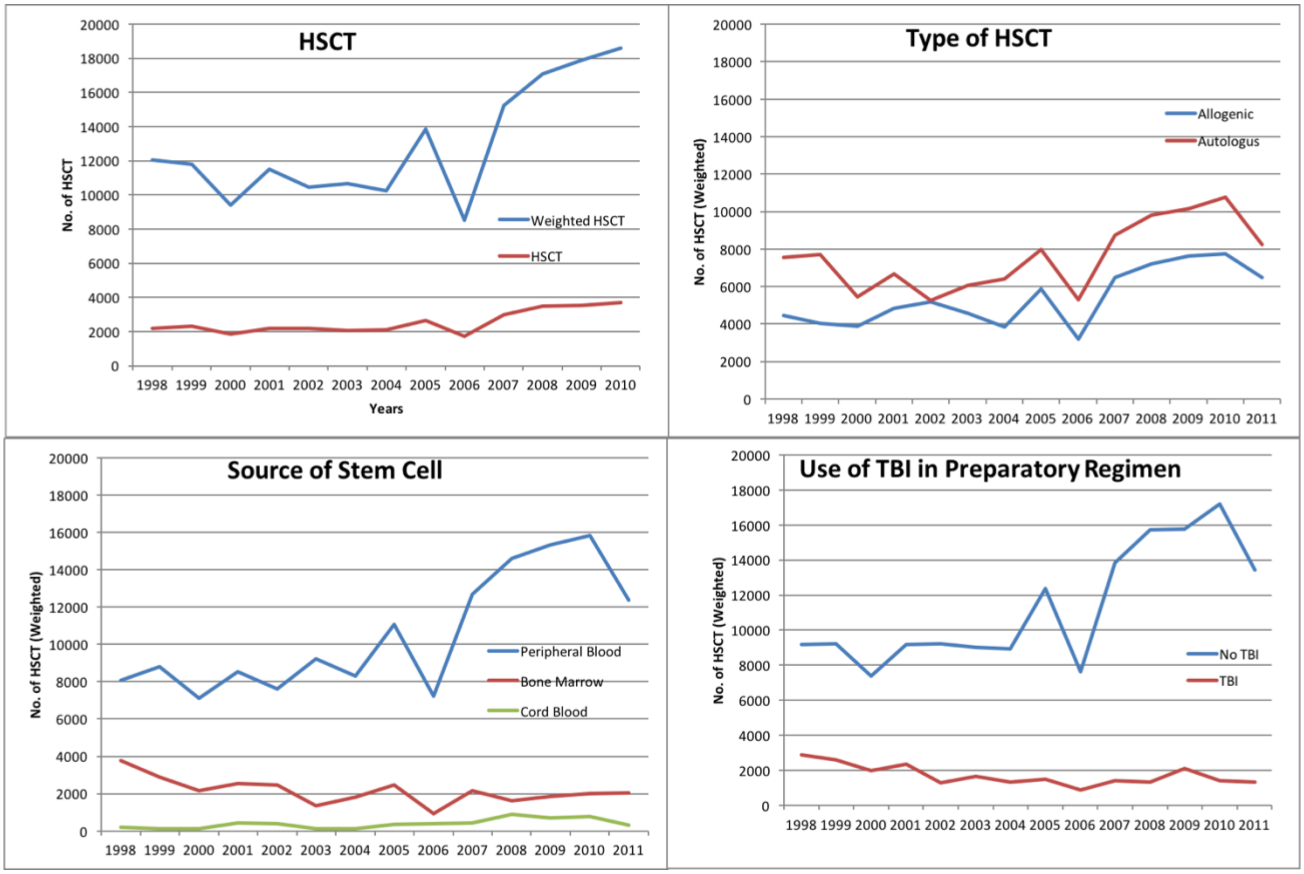

Figure S2: Trends of HSCT, Type of HSCT, Sources of Stem Cells, and Total Body Irradiation in United States from 1998 to 2011.

References:

1 HCUP Nationwide Inpatient Sample (NIS).Healthcare Cost and Utilization Project (HCUP). Edited by: Agency for Healthcare Research and Quality, Rockville, MD; 2007-2009. (http://www.hcup-us.ahrq.gov/nisoverview.jsp)

2 Steiner, C., Elixhauser, A. & Schnaier, J. The healthcare cost and utilization project: an overview. *Eff Clin Pract* **5**, 143-151 (2002).

3 Goel, R. *et al.* Platelet transfusions in platelet consumptive disorders are associated with arterial thrombosis and in-hospital mortality. *Blood* **125**, 1470-1476, doi:10.1182/blood-2014-10-605493 (2015).

4 Jones, J. A. *et al.* In-hospital complications of autologous hematopoietic stem cell transplantation for lymphoid malignancies: clinical and economic outcomes from the Nationwide Inpatient Sample. *Cancer* **112**, 1096-1105, doi:10.1002/cncr.23281 (2008).

5 Deyo, R. A., Cherkin, D. C. & Ciol, M. A. Adapting a clinical comorbidity index for use with ICD-9-CM administrative databases. *J Clin Epidemiol* **45**, 613-619 (1992).

6 Deshmukh, A. *et al.* In-hospital complications associated with catheter ablation of atrial fibrillation in the United States between 2000 and 2010: analysis of 93 801 procedures. *Circulation* **128**, 2104-2112, doi:10.1161/CIRCULATIONAHA.113.003862 (2013).

7 Ananthakrishnan, A. N. & McGinley, E. L. Weekend hospitalisations and post-operative complications following urgent surgery for ulcerative colitis and Crohn's disease. *Aliment Pharmacol Ther* **37**, 895-904, doi:10.1111/apt.12272 (2013).

8 Badheka, A. O. *et al.* Balloon mitral valvuloplasty in the United States: a 13-year perspective. *Am J Med* **127**, 1126 e1121-1112, doi:10.1016/j.amjmed.2014.05.015 (2014).

9 Bergstralh EaK, J. Computerized matching of cases to controls using the greedy matching algorithm with a fixed number of controls per case. Edited by: Mayo Clinic; 2003.

10 Malat, G. *et al.* Kidney donor risk index (KDRI) fails to predict kidney allograft survival in HIV (+) recipients. *Transplantation* **98**, 436-442, doi:10.1097/TP.0000000000000073 (2014).

11 Ananthakrishnan, A. N., McGinley, E. L. & Binion, D. G. Excess hospitalisation burden associated with Clostridium difficile in patients with inflammatory bowel disease. *Gut* **57**, 205-210, doi:10.1136/gut.2007.128231 (2008).

12 Kaplan, G. G. *et al.* Inflammatory bowel disease patients who leave hospital against medical advice: predictors and temporal trends. *Inflamm Bowel Dis* **15**, 845-851, doi:10.1002/ibd.20835 (2009).

13 Nguyen, G. C. & Sam, J. Rising prevalence of venous thromboembolism and its impact on mortality among hospitalized inflammatory bowel disease patients. *Am J Gastroenterol* **103**, 2272-2280, doi:10.1111/j.1572-0241.2008.02052.x (2008).
